# Comparison of Different Hydrocolloids on the Novel Development of Muffins from “Purple Yam” (*Dioscorea alata*) Flour in Sensory, Textural, and Nutritional Aspects

**DOI:** 10.1155/2021/9970291

**Published:** 2021-10-16

**Authors:** Dewni Gunasekara, Ashani Bulathgama, Indira Wickramasinghe

**Affiliations:** ^1^Department of Food Science and Technology, Faculty of Applied Sciences, University of Sri Jayewardenepura, Nugegoda, Sri Lanka; ^2^School of Science, RMIT University, Melbourne, Australia

## Abstract

Hydrocolloids can act as gluten substitutes to form the structural equivalents of the gluten network in gluten-free bakery products. “Purple yam” (*Dioscorea alata)* is one of the underutilized yams in Sri Lanka with high nutritional potential. The overall objective of this study was to develop gluten-free muffins using “Purple yam” (*Dioscorea alata)* flour with hydrocolloids (pectin, xanthan gum, and guar gum) and investigate the nutritional composition and selected properties of the muffins. The texture profiles of gluten-free muffins were analyzed through the following parameters: hardness, adhesiveness, cohesiveness, etc. The chromameter values were obtained and sensory evaluations for gluten-free muffins were carried out. The highest moisture content was recorded in pectin-incorporated muffins (17.70 ± 0.50%). The protein content of all three types of muffins was around 5%. The highest fat content was recorded in pectin-incorporated muffins (19.26 ± 0.51%). The ash content of all three types of muffins was around 2%. Potassium was the most predominant element found in each muffin. The hardness of guar gum-incorporated muffin (6379.3 ± 135.9 g) was greater than that of the pectin-incorporated one (6082.3 ± 23.4 g). Xanthan gum-incorporated muffins had significantly decreased cohesiveness (0.19 ± 0.04). The highest springiness was obtained in pectin-incorporated muffin (37.13 ± 1.61 mm). The descending order of the chewiness of muffin is pectin − added > xanthan gum − added > guar gum − added sample. According to the sensory evaluation, pectin-incorporated muffin was the best as it had obtained the highest sum of ranks for appearance, color, taste, after taste, and overall acceptability.

## 1. Introduction

Celiac disease is a chronic autoimmune disease that causes inflammation of the upper small intestine in genetically predisposed individuals [1]. Nowadays, it is more common in some parts of the world and it seems to be rising gradually [2, 3]. The best treatment of celiac disease is to reduce the consumption of wheat, rye, and barley and use of a gluten-free diet [4, 5]. Considering the important role of gluten-free products in the diet of celiac patients, the quality of these products should be carefully assessed and reviewed. There are many underutilized yam varieties in Sri Lanka with high nutritional potential [6]. Meanwhile, the edible yams belonging to the genus Dioscorea have been used as an energy source for decades [7]. With the urbanization and changing food habits, most of these underutilized yams have lost their significance. *D. alata* cultivars were discovered to provide medicinal benefits, including immunological activation and antihypertensive properties, in addition to their culinary value [8, 9]. Hence, this research attempted to develop gluten-free muffins with the addition of 0.3% (*w*/*w* based on flour) of three different hydrocolloids (pectin, xanthan gum, and guar gum) based on *D. alata* flour. *Dioscorea alata* belongs to the Dioscoreaceae family. *D. atropurpurea* and *D. sativa* are some synonyms [10]. About 600 species of *Dioscorea* are consumed in various parts of the world [11]. Pangyuan [12] has mentioned that *D. alata* has medicinal properties and can be used in Ayurvedic and Western medicine. Purple yam is a great source of carbohydrates, fibre, and potassium and contains antioxidants including anthocyanins and vitamin C [13].

Some of the properties of bakery products such as bread and cake have unique characteristics that are comparatively difficult to replace without the gluten in the product. In recent years, several studies have identified that “hydrocolloids” can act as gluten substitutes to improve the texture, structure, and rheological properties of bakery products [14]. Hydrocolloid is a food additive that has properties that can improve the viscosity of the dough and enhance the characteristics of gluten-free bakery products [15]. For viscosity control and better processing tolerance in cake recipes, hydrocolloids are used today. The industrial-scale cake production involves high-shear mixing. The batter is normally processed through a pumping machine and continuous mixers. Hydrocolloids can control the viscosity of the batter and prevent degassing of the batter during its processing [16]. The commonly used hydrocolloids in the bakery industry are pectin, egg albumin, galactomannans, xanthan gum, Arabic gum, and guar gum [17]. Among these hydrocolloids, the impact on the characteristics of gluten-free muffins was evaluated using three different hydrocolloids including pectin, xanthan gum, and guar gum. These hydrocolloids have been used in the bakery industry for crumb softness and to improve the keeping quality during storage [17, 18].

Pectin is frequently extracted from citrus fruit and apples, and it can be used as a vegan substitute in gluten-free formulas. Pectin is a complex carbohydrate and it improves the structure of breads and cakes. Moreover, pectin promotes moisture retention that keeps baked products from drying out and keeps them soft. Xanthan gum is a nonabsorbing polymer that shows good thickening ability even at low concentrations, and it shows good water holding capacity [19, 20]. A stretchy web is formed when xanthan gum is mixed with water, which is similar to gluten's structure. However, xanthan gum is more expensive. Guar gum is an effective enhancer, as it reduces the stiffness of muffins and increases the specific volume [21]. It is less expensive than xanthan gum but has incredible thickening power. The bakery products formulated with guar gum are less “gummy” than products made with xanthan gum. Both xanthan gum and guar gum have laxative properties, which can cause digestive distress in some people. In addition xanthan gum and pectin improve the softer texture, porosity, and elasticity of the crumb, as well as sensory attributes [22].

Therefore, this study is aimed at assessing the effects that hydrocolloids have on the texture profile, nutritional composition, chromameter values, and sensory attributes.

## 2. Materials and Methods

### 2.1. Ingredients

Fully mature, undamaged *D. alata* yams were collected from the Agricultural Research Station, Department of Agriculture, Telijjawila, Sri Lanka. The other ingredients used were sugar, whole egg, baking powder, sunflower oil, and milk were supplied by the Supermarkets, Wijerama, Sri Lanka. Pectin, xanthan gum, and guar gum were obtained from the suppliers of Colombo, Sri Lanka.

### 2.2. Preparation of Flour Samples

*D. alata* yams were hand peeled, washed, and cut into thin pieces and dried in an air convection oven (MA 035 Marconi, Colombo, Sri Lanka) at 60°C for 24 h. The dried pieces were powdered using a laboratory scale grinder (ABBLBL468AB, Colombo, Sri Lanka) and sifted through a 300 *μ*m sieve. The flour samples were sealed and packed in airtight containers for further preparation.

### 2.3. Development of Gluten-Free Muffins

The gluten-free muffins were developed according to the modified method described by Bhaduri and Navder [23].The muffin recipe was formulated with *D. alata* flour (70 g), sugar (45 g), whole egg (35 g), baking powder (3.5 g), sunflower oil (30 g), and milk (55 ml), and muffins were prepared under four treatments. Three treatments were developed with the incorporation of three different hydrocolloids, and one treatment was developed without the addition of hydrocolloids as a control sample. Those three treatments were as follows: incorporated pectin, xanthan gum, and guar gum by 0.3% (*w*/*w* based on flour) as a replacement for gluten [24]. Also, as a comparison, muffins were prepared without the addition of any hydrocolloid percentage. The dry ingredients *D. alata* flour, baking powder, and pectin/xanthan gum/guar gum were weighed and sifted together. The whole egg, milk, sugar, and sunflower oil were mixed well, and the flour mixture was added and was mixed for 5 min at a speed of 240 rpm in a multifunction food processor (Rowenta Universo 700, France); vanilla was finally added and mixed well. After scraping down the bowl, 50 g of batter was weighed and placed in aluminum baking molds. The muffins were baked in an oven (Indesit built-in electric oven, 2200 W, Colombo, Sri Lanka) at 180°C for 30 min [25, 26].

### 2.4. Proximate Analysis

The proximate analysis was carried out to determine the moisture content (AOAC 931.04), crude protein content (AOAC 920.87), total fat content (AOAC 922.06), and total ash content (AOAC 923.03) of each muffin samples [27]. The results were expressed on dry weight (DW) basis and all measurements were performed in triplicates. The determination of the mineral (potassium, calcium, iron, zinc, magnesium, and copper) content was carried out according to the AOAC official procedure 975.03 method of dry ashing followed by the atomic absorption spectroscopy method [27]. The atomic absorption spectroscopy machine used was Hitachi model 170-10, and the muffle furnace (Wisetherm, Colombo, Sri Lanka) was used to get ash samples.

### 2.5. Crust and Crumb Color

The surface crust and crumb color of muffins, represented by the CIE *L*∗*a*∗*b*∗ model (*L*∗:lightness; *a*∗:redness; *b*∗:yellowness), were measured by using a chromameter (Lovibond® LC100, Colombo, Sri Lanka). Color values were taken as replicates (*n* = 3) in different areas of the crumb and crust on the muffin surface as suggested by Broyart et al. [28].

### 2.6. Texture Characterization

For two-cycle compression, a CT3 texture analyzer (50 kg, Brookfield, USA) was used to measure the force-time curves as penetration profiles. The method for texture profile analysis was used as recommended in Brookfield's instruction manual. The texture analyzer was supplied with a load cell of 100 g and application software (Brookfield Texture PRO CT) [29]. Two successive compressions were carried out on each sample. The resulting force-time curves were developed for hardness, chewiness, gumminess, adhesiveness, and cohesiveness. The accessory used for all measurements was a TA11/1000 (25.4 mm diameter cylinder probe, stainless steel, 10 g) probe. Each sample was tested two times at the test and a return speed of 1 mm/sec and a target depth of 2 mm. Trigger load was 1.0 g, the pretest speed was 2 mm/sec, and the data rate was 10 points/sec. Upon two compression cycles, the probe was automatically returned to the initial starting point and the texture analyzer was reset for the next test. All analyses were conducted at ambient temperature. The force-time deformation curves during compression and decompression cycles were obtained each time. The same textural properties were measured for all prepared samples. All results were expressed in a report with values automatically calculated by the analyzer's software [30, 31].

### 2.7. Sensory Evaluation

The three types of muffins were subjected to evaluate their appearance, color, aroma, texture, taste, after taste, and overall acceptability by 30 members of a semitrained preference test panel from the Department of Food Science and Technology, University of Sri Jayewardenepura. Panelists were informed that they would be evaluating gluten-free muffins, and they were presented with three code numbers (coded “000”). The order of presentation of the gluten-free muffins was also random. The panelists were asked to evaluate the samples according to their preferences. Samples were evaluated using a 5-point hedonic scale, with 1 for “dislike extremely” and 5 for “like extremely” [23]. They were also instructed to rank the products in the order in which they liked them, with 1 for “least liked” and 3 for “most liked.” They were also asked how often they ate muffins, if they had tried gluten-free products before and if anyone in their family had celiac disease. Water and unsalted crackers were provided to panelists to cleanse their palates between samples.

### 2.8. Statistical Analysis

The statistical analysis of data was carried out for all experiments using ANOVA to test the significance of each variable (*a* = −0.05) and followed by comparisons performed using the Tukey test by the statistical software MINITAB 17.

## 3. Results and Discussion

**Figure 1:**
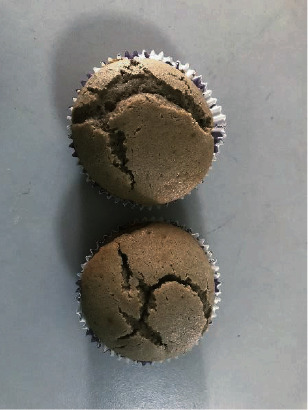
*D. alata* flour muffins without the addition of hydrocolloids.

**Figure 2:**
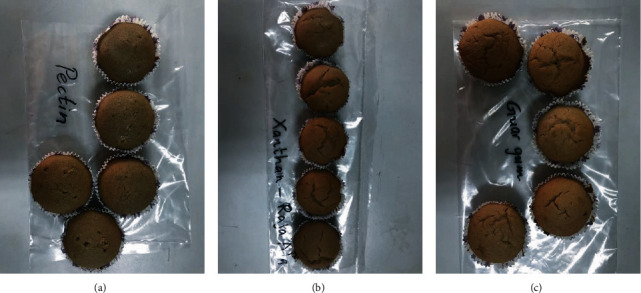
*D. alata* flour muffins. (a) Pectin-incorporated muffins. (b) Xanthan gum-incorporated muffins. (c) Guar gum-incorporated muffins.

By incorporating pectin, xanthan gum, and guar gum, three types of gluten-free muffins were developed with *D. alata* flour (Figure 1). Also, as a comparison, control muffins were prepared without the addition of hydrocolloids. Images of the muffins are presented in Figure 2. The muffins developed with 100% of *D. alata* flour without the addition of any hydrocolloid percentage that appeared in a dry and extreme tough texture and gave inedible characteristics. Also, the preparation of these muffins was hard due to the toughness of the mixture. According to Figure 2, the muffins' top was highly cracked when compared with hydrocolloid-incorporated muffins. Due to the inedible characteristics showed in *D. alata* flour muffins without the addition of any hydrocolloid percentage, further analysis was only carried out with the hydrocolloid-incorporated muffins. Three types of hydrocolloid-incorporated muffins were highly appreciated by the sensory panel because they had got the characteristic taste of purple yam flour and they were similar to traditional wheat flour muffins with their appearance and soft texture.

The results obtained from the proximate analysis of three muffin samples are shown in Table 1. Some previous studies have expressed the average moisture content of muffins which is 33.0%, and Lostie [32] has stated that a typical cake has a moisture content between 15 and 30%. Therefore, a slight decrease in moisture content was observed in all treatments. The highest moisture content was recorded in pectin-incorporated muffins. It might be due to the pectin being a gelling-type hydrocolloid [33]. Furthermore, pectin has the highest water holding capacity (57 g water/g) when compared to xanthan gum and guar gum [34]. Dogan et al. [35] have identified that the water absorption capacity (WAC) of guar gum is higher than that of xanthan gum. However, some studies have evaluated that the water holding capacity of xanthan gum (19.2 g water/100 g hydrocolloid) is higher than that of guar gum (4.8 g water/100 g hydrocolloid) [36]. Hydrocolloids vary their properties, due to the origin, the variety, and the lifetime of the plant if it is a plant extraction and the extraction method. Therefore, these different properties of hydrocolloids cause different values of water holding capacity. In this study, there was no significant difference in moisture content between the xanthan gum muffins and the guar gum muffins. The highest fat content was recorded in pectin-incorporated muffins (19.26 ± 0.51%), and the lowest was recorded in xanthan gum-incorporated muffins (18.62 ± 0.25). The fat content did not show any significant difference between the three types of muffin samples (*p* < 0.05). The protein content of the three types of muffins ranged from 5.38% to 5.49%, and the total ash content was around 2%. There was no significantly different protein and ash content between all muffin samples according to the one-way ANOVA Tukey pairwise comparison test at a 0.05 significance level. Potassium (K) was the most predominant element found in each muffin. Also, the main mineral in purple yam is potassium (K). 100 g of cooked purple yam provides 13.5% of the daily value (DV) of potassium [37].

The color values (*L*, *a*∗, and *b*∗) of the crumb and crust of muffins were given in Table 2, and for the *a*∗ value, a negative value indicates the closeness to green and a positive value indicates the closeness to red color [38]. Additionally, a negative *b*∗ value indicates the closeness to blue and a positive value indicates the closeness to the yellow color as all products only show positive values and *b*∗>*a*∗ indicates that all products are closer to the yellow color. The lightness of the crumb of the muffin which was prepared by incorporating xanthan gum has significantly the highest lightness when compared with the other two muffins' crumbs. The crumb of the pectin-incorporated sample has the significantly lowest lightness. The surface crust color is one of the critical quality attributes, because it is directly responsible for the initial consumer's acceptance [39]. The crust color is a result of browning reactions: combined Maillard reaction and sugar caramelization. When the surface temperature reaches 100°C, browning reactions are initiated, and then, the crust turns darker [28].

There was not a significant difference between the crust of xanthan gum-incorporated muffin and the crust of guar gum-incorporated muffin. The crust of the pectin-incorporated muffin has shown the highest darkness. The *a*∗ values of the crumb of all three types of muffins did not show a significant difference. In addition, the *b*∗ values of both the crumb and the crust did not show a significant difference at a 0.05 significant level.

The primary texture profile analysis (TPA) parameters such as hardness, adhesiveness, springiness, and cohesiveness and secondary parameters of gumminess and chewiness are shown in Table 3. Hardness is defined as the maximum peak force during the first compression cycle (first bite); here, the highest hardness was shown in xanthan gum-incorporated muffin. The hardness of guar gum-incorporated muffin (6379.3 ± 135.9 g) was greater than pectin-incorporated muffin (6082.3 ± 23.4 g). Adhesiveness is the work necessary to overcome the attractive forces between the surface of the food and the surface of other materials with which the food comes into contact (e.g., tongue, teeth, and palate) [23]. Based on ANOVA instrumental texture analysis, significant differences in adhesiveness of the samples were obtained. There was no significant difference of adhesiveness between the three types of muffins at 0.05 significant level. Cohesiveness is defined as the ratio of the positive force during the second compression to that during the first compression, and this parameter is the strength of the internal bonds which make up the body of the product. Xanthan gum-incorporated muffins had significantly decreased cohesiveness (0.19 ± 0.04) compared to the other muffins, and lower compression energy was required. Typically, a more cohesive product retains more gas and has a higher volume [40]. Springiness is related to the height that the food recovers during the time that elapses between the end of the first bite and the start of the second bite. Springiness is associated with freshness in a product with a high-quality muffin having higher springiness values [41]. The highest springiness was obtained in pectin-incorporated muffin (37.13 ± 1.61 mm). Moreover, xanthan gum-incorporated muffin was significantly springier than any guar gum-incorporated muffins. There was no significant difference in the gumminess of the xanthan and guar gum muffins. The decreasing order of chewiness of muffin is pectin − added > xanthan gum − added > guar gum − added sample.

A sensory evaluation was carried out to identify the consumer acceptability of the muffins and to select the best suitable hydrocolloid for *D. alata* muffins. The effect of the sensory properties such as appearance, color, aroma, texture, taste, after taste, and overall acceptability was evaluated among the three different muffins. Nonparametric data obtained from this sensory evaluation were statistically analyzed by using the Friedman test at a 95% confidence level. The mean separations were done by using the Friedman test at a 95% confidence level. The three samples were named as sample number 319: xanthan gum-incorporated muffin, sample number 482: pectin-incorporated muffin, and sample number 593: guar gum-incorporated muffin.

**Figure 3:**
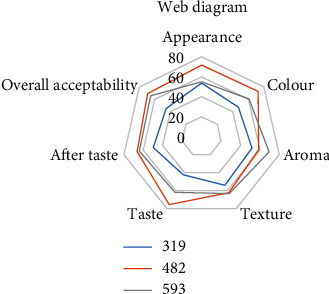
Web diagram for the sensory evaluation of the three muffin samples.

The sensory characteristics of gluten-free muffins depend on the number and variety of hydrocolloids used as a gluten substitute, as this influences the interaction between them and starch, which is the key ingredient in the dough [42]. The web diagram for the sensory evaluation of the three muffin samples is shown in Figure 3. According to the sensory evaluation, pectin-incorporated muffin was the best as it had obtained the highest sum of ranks for appearance, color, taste, after taste, and overall acceptability. Furthermore, pectin-incorporated muffins had obtained a medium sum of ranking values of aroma and texture. Pectin is a complex carbohydrate used to thicken jams and jellies. Dried pectin, which can be difficult to find, helps provide structure for breads and cakes [43]. It absorbs moisture, which helps keep baked goods from drying out and keeps them soft [44]. Therefore, it can be concluded that muffins are very soft products and because of the hygroscopic ability of pectin, pectin-incorporated muffins stayed fresher than other muffins. Moreover, pectin was obtained from a natural source: leaves of “*Cylea peltata*” (Kahipiththan tree). Guar gum is made from a legume plant. It is less expensive than xanthan gum but has incredible thickening power. It makes muffins that are less “gummy” than muffins made with xanthan gum. Both xanthan gum and guar gum have laxative properties, which can cause digestive distress in some people. Older gluten-free recipes relied heavily on xanthan gum [45]. Xanthan gum is made from corn. Xanthan gum is used for only a trace amount in recipes; if not, the product can become heavy or slimy [46]. Gums form a structural equivalent of a gluten network when mixed with water. However, xanthan gum is expensive and some people who are sensitive to gluten are also sensitive to xanthan gum. Some people can taste the gum in baked goods.

## 4. Conclusions

The hydrocolloids influence the moisture content of gluten-free muffins. The highest moisture content was recorded in pectin-incorporated muffins (17.70 ± 0.50%). Moreover, the chromameter values and texture profile of muffins varied according to different hydrocolloids. The hardness of xanthan gum-incorporated muffin (6963.3 ± 130.5 g) was the highest. Xanthan gum-incorporated muffins had significantly decreased cohesiveness (0.19 ± 0.04) compared to the other muffins. The highest springiness was obtained in pectin-incorporated muffin (37.13 ± 1.61 mm). According to the sensory evaluation, pectin-incorporated muffin was the best as it had obtained the highest sum of ranks for appearance, color, taste, after taste, and overall acceptability.

## Figures and Tables

**Table 1:** Nutritional composition of three different muffins.

Parameters	Pectin-incorporated muffins	Xanthan gum-incorporated muffins	Guar gum-incorporated muffins
Moisture %	17.70 ± 0.50^a^	14.16 ± 0.43^b^	13.88 ± 0.81^b^
Protein %	5.42 ± 0.38^a^	5.38 ± 0.18^a^	5.49 ± 0.06^a^
Fat %	19.26 ± 0.51^a^	18.62 ± 0.25^a^	18.82 ± 0.30^a^
Ash %	2.07 ± 0.04^a^	2.07 ± 0.04^a^	2.11 ± 0.04^a^
K	431.55 ± 6.84^a^	451.68 ± 15.02^a^	419.3 26.3^a^
Mg	35.61 ± 1.54^b^	49.51 ± 0.54^a^	50.74 ± 0.56^a^
Ca	6.04 ± 0.45^c^	7.38 ± 0.66^b^	9.14 ± 0.31^a^
Zn	1.01 ± 0.03^a^	1.16 ± 0.04^a^	1.18 ± 0.11^a^
Cu	0.41 ± 0.01^b^	0.458 ± 0.01^a^	0.43 ± 0.01^b^
Fe	0.93 ± 0.06^a^	1.04 ± 0.04^a^	1.01 ± 0.05^a^

Values in the same row with different superscripts indicate significant difference (*P* < 0.05).

**Table 2:** Average chromameter values for three different muffins.

Muffin variety		*L*∗	*a*∗	*b*∗
Pectin incorporated muffin	Crumb	35.93 ± 0.85^c^	7.10 ± 1.47^d^	16.27 ± 1.10^a^
	Crust	37.30 ± 0.20^e^	08.40 ± 0.92^c^	16.70 ± 1.49^b^
Xanthan gum incorporated muffin	Crumb	48.50 ± 0.30^b^	6.97 ± 0.12^d^	18.33 ± 1.23^a^
	Crust	38.70 ± 0.27^d^	11.50 ± 0.99^b^	17.07 ± 5.25^b^
Guar gum incorporated muffin	Crumb	45.40 ± 0.20^a^	6.13 ± 1.27^d^	18.07 ± 0.76^a^
	Crust	39.30 ± 0.61^d^	14.10 ± 0.44^a^	19.63 ± 8.00^b^

^a,b,c,d,e^Values in the same column with different superscripts indicate significant differences (*p* < 0.05).

**Table 3:** Instrumental texture analysis of muffins.

Parameters	Pectin-incorporated muffin	Xanthan gum-incorporated muffin	Guar gum-incorporated muffin
Hardness (g)	6082.3 ± 23.4^c^	6963.3 ± 130.5^a^	6379.3 ± 135.9^b^
Adhesiveness (mJ)	11.87 ± 0.55^a^	12.80 ± 0.70^a^	12.37 ± 0.06^a^
Cohesiveness	0.35 ± 0.04^a^	0.19 ± 0.04^b^	0.25 ± 0.04^b^
Springiness (mm)	37.13 ± 1.61^a^	29.76 ± 0.60^b^	24.39 ± 1.92^c^
Gumminess (g)	1849.3 ± 151.7^a^	975.7 ± 97.8^b^	1022.7 ± 89.1^b^
Chewiness (mJ)	657.27 ± 16.90^a^	599.9 ± 19.2^b^	583.57 ± 14.00^b^

^a,b,c^Values in the same row with different superscripts indicate a significant difference (*p* < 0.05).

## Data Availability

The data used to support the findings of this study are included in the article.
